# Revisiting the Examination of Sharp/Dull Discrimination as Clinical Measure of Spinothalamic Tract Integrity

**DOI:** 10.3389/fneur.2021.677888

**Published:** 2021-07-01

**Authors:** Laura Heutehaus, Christian Schuld, Daniela Solinas, Cornelia Hensel, Till Kämmerer, Norbert Weidner, Rüdiger Rupp, Steffen Franz

**Affiliations:** Spinal Cord Injury Center, Heidelberg University Hospital, Heidelberg, Germany

**Keywords:** neurological examination, sensory function assessment, spinothalamic tract, sharp/dull discrimination, pin-prick, spinal cord injury, interrater reliability, ISNCSCI

## Abstract

**Objective:** Revisiting the sharp/dull discrimination as clinical measure of spinothalamic tract function considering the International Standards for Neurological Classification of Spinal Cord Injury (ISNCSCI). Three clinically relevant factors were evaluated as to their impact on reliability: (1) the localization of dermatomes in relation to the sensory level, (2) the examination tool, and (3) the threshold of correct answers for grading of a preserved sharp/dull discrimination.

**Design:** Prospective monocentric psychometric study.

**Setting:** Spinal Cord Injury Center, Heidelberg University Hospital, Germany.

**Participants:** Convenient sample of 21 individuals with subacute spinal cord injury (age: 31–82 years) and 20 individuals without spinal cord injury (age: 24–63 years).

**Assessment:** All participants underwent three assessments for sharp/dull discrimination, applying five commonly used examination tools in seven dermatomes, performed by three trained examiners under conditions in accordance with ISNCSCI.

**Main Outcome Measures:** Assessment of interrater reliability by determining both the Fleiss kappa (κ) coefficient and the percentage agreement between raters. Data were dichotomized regarding the ISNCSCI threshold.

**Results:** Interrater reliability in individuals with SCI was overall substantial (κ = 0.68; CI 0.679–0.681) and moderate (κ = 0.54; CI 0.539–0.543) in dermatomes below the sensory level. All applied tools led to at least moderate reliability below the sensory level (lowest κ = 0.44; CI 0.432–0.440), with the officially endorsed safety pin achieving the highest (substantial) reliability (κ = 0.64; CI 0.638–0.646). Percentage agreement differed between non-SCI (97.3%) and formally intact above level dermatomes in SCI (89.2%).

**Conclusions:** Sharp/dull discrimination as a common clinical examination technique for spinothalamic tract function is a reliable assessment. Independent from the used examination tools, reliability was substantial, with the medium-sized safety pin delivering the most favorable results. Notwithstanding this, all other tools could be considered if a safety pin is not available. Regarding interrater reliability and guessing probability, a threshold of 80% correct responses for preserved sharp/dull discrimination appears to be most suitable, which is in line with current clinical approaches and ISNCSCI. The causal attribution of the identified differences in sharp/dull discrimination between clinically intact dermatomes of individuals with SCI and unaffected dermatomes of individuals without SCI requires future work.

**Clinical Trial Registration Number (German Clinical Trials Register):** DRKS00015334 (https://www.drks.de).

## Introduction

Specific lesion patterns of sensory tract systems are being discussed to play a relevant role regarding the occurrence of neuropathic pain as a common secondary complication of neurological disorders like polyneuropathy or spinal cord injury (SCI) (1–5). In this regard, the most relevant tracts are the lemniscal (epicritic sensibility) and spinothalamic (protopathic sensibility) tracts. For testing the integrity of these tracts in clinical routine, frequently used techniques are the two-point discrimination or light touch sensation to assess the lemniscal tract system and the pin-prick examination to evaluate the spinothalamic tract function (6–8). The pin-prick examination conceptually contains two consecutive steps: firstly, evaluation of the ability to reliably discriminate between a sharp/pain and dull/pressure sensation, henceforth referred to as sharp/dull discrimination. In case sharp/dull discrimination is intact, the pin-prick sensation is graded by the patient as normal or altered (9, 10). For the pin-prick exam, a safety pin is typically used applying sharp stimuli with its sharp end and dull stimuli with its blunt end (11).

The comprehensive clinical assessment of the sensory tract integrity is particularly essential for characterizing neurological dysfunction after SCI. It is routinely performed in a rigid fashion as part of the standardized neurological examination according to the International Standards for Neurological Classification of Spinal Cord Injury (ISNCSCI) (11). The ISNCSCI assessment quantifies neurological impairments of both motor and sensory function including lemniscal and spinothalamic tract function.

High-quality psychometric properties are an indispensable requirement for any clinical examination technique (12). The general psychometric properties of ISNCSCI are well-investigated, and it is considered to be a “reliable, valid, and responsive instrument for descriptive and evaluative purposes in the adult SCI population” (13). However, the reliability of the pin-prick examination, which includes both the evaluation of the ability to correctly discriminate between sharp and dull and the grading of pin-prick sensation, has been disputed (14). The subjective grading of pin-prick sensation as normal or altered is inherently susceptible to being confounded by multiple factors and, with this, negatively impacting the psychometric properties of the overall pin-prick examination. In contrast, the sharp/dull discrimination represents the rather objective part of the pin-prick examination, given the fact that at least the respective stimuli are applied in a standardized fashion controlled by the examiner only. In addition, the guessing probability can be lowered with a higher number of repetitions. Thus, the results of this part of the examination are less susceptible to direct influence by the tested subject—intentionally or unintentionally. Nevertheless, certain factors of the sharp/dull discrimination as essential part of the pin-prick examination may also compromise reliability and thus need to be quantified:

1. Previous studies did not discriminate between neurologically unimpaired and impaired skin areas, which could have resulted in an overestimation of the reliability of the sharp/dull discrimination (15–19).
2. Different examination tools such as safety pins of different sizes, the Neurotip® examination pin, cotton tips, or devices commonly used for transferring sterile fluids (e.g., Transofix^®^) are applied to test sharp and dull sensations, which could affect the reliability of the sharp/dull discrimination.
3. In cases with generally impaired sensory function, there is the risk of obtaining inaccurate responses due to guessing. Consequently, a well-founded threshold on the required number of correct responses for distinguishing between an intact or absent sharp/dull discrimination is needed.

Therefore, the objective of this study was to investigate the interrater reliability of the sharp/dull discrimination as essential and rather objective part of the pin-prick examination in adults with SCI. This was done in dependency of different examination tools and complemented by revisiting the number of repetitions required for reliable sharp/dull discrimination. The approach of testing in individuals with SCI allowed for two distinct reliability analyses of sharp/dull discrimination: Firstly, a comparison of sharp/dull discrimination between intact dermatomes above and dermatomes with altered sensation below the lesion, and, secondly, a comparison of intact dermatomes between individuals with SCI and non-disabled study participants.

## Materials and Methods

### Study Design

This prospective monocentric psychometric study was designed and conducted at the Spinal Cord Injury Center, Heidelberg University Hospital, Germany. The study protocol was approved by the ethics committee of the Medical Faculty Heidelberg, Germany (S-304/2017) (20), reported to the German Clinical Trials Registry (DRKS00015334) and complies with the “SPIRIT” rules: “Defining standard protocol items for clinical trials” (20). The structure of the manuscript is in accordance with the guidelines for reporting reliability and agreement studies (GRRAS) (21). To ensure current quality standards, all raters were trained within the European Multicenter about Spinal Cord Injury (EMSCI) (22–24) network according to the 7^th^ ISNCSCI edition updated in 2015 (25).

### Recruitment

Participants were recruited from the in-patient cohort by convenience sampling and gave written informed consent prior to study inclusion. The recruitment period was from August 2017 to February 2019. Eligibility for the study required full legal age (≥18 years) and the ability to consent. Recruitment was done in two different groups of 20 participants each, either without (non-SCI) or with SCI. In the SCI group, participants with subacute complete or incomplete SCI (≥12 weeks post injury) and any neurological level of injury were included. Non-disabled controls were included as a reference for determining the characteristics of sharp/dull discrimination in neurologically intact dermatomes. Exclusion criteria for both groups comprised skin diseases in the designated dermatomes, multidrug-resistant germs, major brain and/or peripheral nervous system injury/disease, relevant psychiatric disorders or cognitive impairment, and any other condition involving an impact on the ISNCSCI and sharp/dull discrimination.

### Study Protocol

Prior to study inclusion, individuals with SCI received an ISNCSCI (11, 25, 26) examination for sensory-level determination, performed by the same examiner (LH). Each study participant of the non-SCI and SCI groups underwent three rounds of sharp/dull discrimination testing at the ISNCSCI key sensory points in seven predefined dermatomes (C5/T1/T4/T10/L4/L5/S1) conducted by three different raters. The selection of dermatomes should represent all spinal regions (cervical/thoracic/lumbar/sacral). Moreover, body regions with different characteristics, such as more or less haired skin, differently pronounced subcutaneous tissue, and protuberances, should be included. This approach was chosen instead of a full ISNCSCI examination to shorten the assessment and thereby reduce the burden to participants. The study examination was not only done with the 4-cm safety pin recommended by ISNCSCI but also with four additional examination tools, based on a survey conducted in 2015 among all active EMSCI centers (23)—in detail, a larger safety pin (5 cm of length), the transfer spike “Transofix^®^” (B. Braun Melsungen AG, Melsungen, Germany), the neurological examination pin “Neurotip^®^” (Owen Mumford Ltd., Woodstock, UK), and a broken cotton tip with wooden handle (length 15 cm, diameter 1 cm). A cannula for peripheral venous catheters, which was reported to be used for testing sharp/dull discrimination, was omitted due to the risk of causing harm to the study participants.

Each rater tested all seven dermatomes and all tools in a random order on a randomly selected side of the body. This resulted in five iterations of testing per participant, dermatome, and rater. In each session, each dermatome was tested more frequently than in clinical routine (60 applied stimuli per dermatome). A (hyper-)sensitization of dermatomes due to multiple stimuli was considered negligible due to a randomized order of the examined dermatomes per tool. The sharp/dull discrimination as part of the pin-prick examination recommended by ISNCSCI, which is optimized for bedside use, allows a varying number of repetitions depending on the examiner's clinical judgment. In contrast, the present study design used a fixed number of applied stimuli per examination tool. The sharp/dull discrimination examination consisted of 12 stimuli per dermatome, six times sharp and six times dull in random sequence, representing a block randomization with a block size of 4. It was recorded whether the type of stimulus was identified correctly or incorrectly. An unperceived stimulus led to an incorrect rating. A detailed description of the differences between the study setting, the common neurological examination, and ISNCSCI is illustrated in Supplementary Figure 1.

To instruct and define an intact reference, the utilized examination tool was initially applied to the cheek of the participant who was not blinded at that time (11). During the testing, participants were in supine position and blinded to the applied stimuli. Each study participant was tested in a quiet environment in three sessions by three different raters: one board-certified physiatrist (CH), two postgraduate trainees (TK/DS), and one physical therapist (LH). Upon completion of the studies on non-SCI individuals, the study team changed before participants with SCI were included. Hence, TK examined all non-SCI participants and CH all individuals with SCI. LH and DS examined all participants of both groups. Given the fact that both groups of participants were tested by only the same assessors, we expected only a minor impact on interrater reliability in data analysis. The time interval between each session had to be at least 1 day and should not exceed 7 days. Raters and participants were requested to refrain from exchanging information with other patients or within the clinical team about the study examinations and the used tools.

Reliability studies should consider the risk of behavioral changes in individuals while participating in a study, a phenomenon known as the Hawthorne effect (21, 27). However, we assumed that the Hawthorne effect did not have a major impact on the results of the study, given the fact that the study-related assessments are part of the routinely applied clinical examination and thereby well known to the participants with SCI.

### Outcome Measures and Statistical Analysis

The numbers of correct responses following 12 applied stimuli per participant, dermatome, examination tool, and rater were recorded in an in-house-developed software written in Visual Basic for Applications. Microsoft^®^ Excel (Microsoft^®^ Corporation) was used as graphical user interface to perform the randomization (dermatome and examination tool order, side of the body, sequence of sharp/dull stimuli applications). The data were initially stored as xlsx files and subsequently processed and analyzed in SPSS^®^ 26 (IBM^®^).

The number of correct responses was dichotomized based on the ISNCSCI rules considering an 80% threshold for correct responses. Thus, for the 12 applied stimuli per dermatome and tool, a theoretical number of at least 9.6 correct responses would lead to a rating as preserved distinction between sharp and dull. For practical reasons, a rate of at least 10 correct responses out of 12 was considered as intact sharp/dull discrimination. Less than 10 correct responses to the 12 stimuli were consequently considered as absent sharp/dull discrimination. This dichotomized response rate is henceforth referred as variable “Correct Responses Binary” (CR2).

Subsequently, Fleiss kappa coefficients (Fleiss κ) of CR2 were determined as the primary endpoint for interrater reliability. Fleiss κ is non-weighted, corrected for chance, and applicable to three raters, small sample sizes, and nominal-scale data. κ varies from −1 to +1 whereby a positive value indicates that the agreement is better than an expected chance agreement (28, 29). The strength of agreement is appraised as “moderate” for κ 0.41–0.60, as “substantial” for κ 0.61–0.80, and as “almost perfect” for κ 0.81–1.0. Values below 0.41 are appraised as “fair” (κ 0.21–0.40), “slight” (κ 0.00–0.20), or “poor” (κ <0.00) (30). As required for appropriate interpretation and comparability, confidence intervals (CI) were reported for each κ (31, 32).

Studies (16, 18, 31, 33) concerning psychometric properties of the ISNCSCI pin-prick examination found moderate to substantial interrater reliability based on total scores. In our study, however, the assumed effect of an overestimation of reliability by mixing results of non-intact and intact dermatomes in total scores cannot be predicted. Consequently, we expected slightly inferior but still moderate reliability in our sensory-level adjusted design.

In dermatomes AT/ABOVE the sensory level and of non-SCI participants, CR2 is skewed (34) toward correct results. This ceiling effect (35) finally led to the problem, that κ was not interpretable, which is a known problem of reliability coefficients corrected for chance (34). Accordingly, another endpoint was necessary to enable a comparison between groups. Thus, percent agreement of CR2 between raters was chosen as secondary endpoint, because it is also known as a “more intuitive measure for clinical practice” (19).

### Subgrouping

The outcome variable CR2 was reported on the examined side of the body for the SCI cohort as a whole as well as grouped in reference to BELOW and AT/ABOVE the sensory level. The examination tools were handled as a further grouping variable.

**Figure 1:**
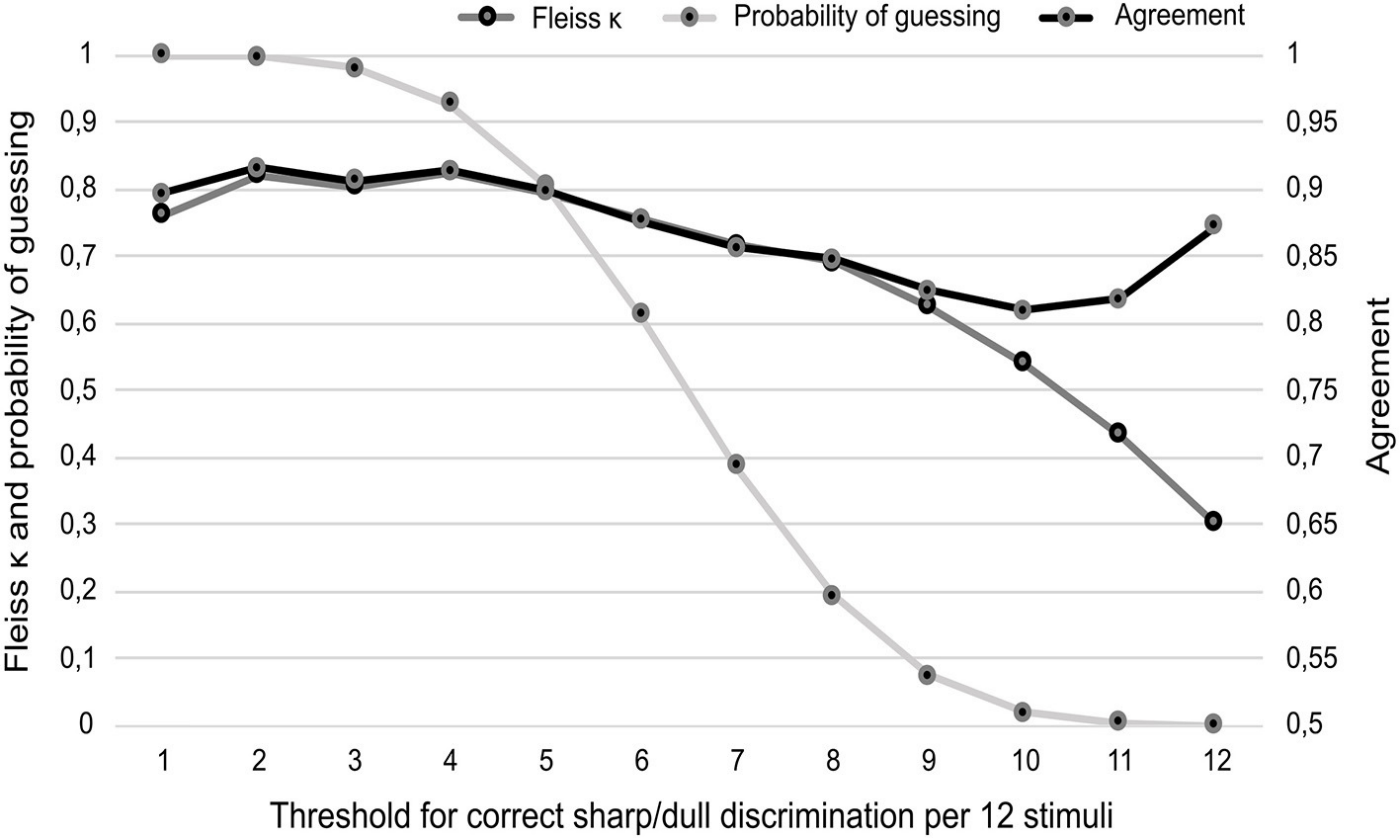
Results of percentage agreement and interrater reliability for three raters in due consideration of the probability of guessing the correct result (vertical axes). The different thresholds in terms of classification of an intact sharp/dull discrimination are given on the (horizontal axis). Data collection was based on aliquot random repetition of in total 12 sharp and dull stimuli. For the sake of interpretability, the scaling of the vertical axes is arranged in a ratio of 1:2 for Fleiss κ/ probability of guessing and percentage agreement. Agreement is presented as decimal fraction of the percentage.

The 80% threshold for correct responses was investigated by comparing all other possible thresholds in this experimental setup with a fixed number of 12 repetitions. The cutoff value for dichotomization was systematically analyzed. Accordingly, it was gradually increased by 1 to evaluate each dichotomization from 1 up to 12 correct responses. The percentage agreement, the interrater reliability (Fleiss κ), and the probability of guessing was taken into account (Figure 1). The guessing probability was calculated by the cumulative distribution function of a binomial distribution (guessing probability per stimuli *p* = 0.5).

## Results

The characteristics of 20 non-disabled controls (non-SCI group) and 21 individuals with SCI (SCI group) are shown in Table 1. Both groups had a comparable, slightly elevated body mass index (SCI: 28.1 ± 5.8 kg/m^2^; non-SCI: 25.7 ± 2.9 kg/m^2^, *p* = 0.66) but differed in age (SCI: 58.8 ± 14.3 years; non-SCI: 40.0 ± 10.9 years, *p* < 0.01). In the SCI cohort, the majority had a thoracic lesion (67%) and was motor incomplete (76%). The time after injury ranged from 74 days to 51.2 years (mean 5.3, *SD* 14.3 years). One SCI participant completed only two of the intended three examinations due to an early discharge. Therefore, 21 instead of 20 participants were included in this cohort to generate 20 complete datasets for the determination of interrater reliability. Overall, 287 dermatomes (seven dermatomes per 41 participants), 147 in the SCI group and 140 in the non-SCI group, were tested. Among individuals with SCI, 52 (35.4%) tested dermatomes were AT/ABOVE and 95 (64.6%) BELOW the sensory level.

**Table 1:** Demographics and sample characteristics.

**Characteristics**	**Dimension**	**Individuals with SCI**	**Individuals without SCI**	***p*-value**
Gender	*Male*	12	12	0.58
	*Female*	9	8	
Age	*Year*	58.8 ± 14.3	40.0 ± 10.9	<0.01
Body height	*cm*	171 ± 13.6	176.5 ± 7.1	0.66
Weight	*kg*	83.1 ± 21.8	78.4 ± 9.7	0.66
Body mass index	*kg/m^2^*	28.1 ± 5.8	25.7 ± 2.9	0.17
ASIA Impairment Scale	*A*	5	*n. a*.	*n. a*.
	*B*	1	*n. a*.	*n. a*.
	*C*	8	*n. a*.	*n. a*.
	*D*	7	*n. a*.	*n. a*.
Cause	*Traumatic*	7	*n. a*.	*n. a*.
	*Degenerative*	6	*n. a*.	*n. a*.
	*Ischemic*	4	*n. a*.	*n. a*.
	*Spondylodiscitis*	3	*n. a*.	*n. a*.
	*Neoplasms*	1	*n. a*.	*n. a*.
Neurological level of injury	*Cervical*	6	*n. a*.	*n. a*.
	*Thoracic*	14	*n. a*.	*n. a*.
	*Lumbar*	1	*n. a*.	*n. a*.
Sensory level on examined	*Cervical*	5	*n. a*.	*n. a*.
side of the body	*Thoracic*	15	*n. a*.	*n. a*.
	*Lumbar*	1	*n. a*.	*n. a*.
Time since injury	*Years*	5.3 ± 15.2	*n. a*.	*n. a*.
Examined side of the body	*Right*	11	12	0.53
	*Left*	10	8	
Time between initial ISNCSCI and first study examination	*Days*	5.3 ± 4.1^‡^	*n. a*.	*n. a*.
		9.9 ± 19.2^‡‡^		
Mean time between study examinations	*Days*	5.1 ± 0.62^†^	7.6 ± 0.32	*n. a*.
		8.3 ± 0.44^††^		

*Time intervals between the initial ISNCSCI examination and the first study examination of two individuals with SCI were beyond 14 days (20 and 93 days). Related data is presented with (‡‡) and without regard to major outliers (‡). The mean time intervals between study examinations were within the limits of 7 days, except for six individuals with chronic SCI (4 × 12–16, 1 × 55, and 1 × 88 days). Deviations of protocol-related time limits were due to necessary clinical interventions and general clinical conditions. Related data is presented with (††) and without outliers (†). ASIA, American Spinal Injury Association; ISNCSCI, International Standards for Neurological Classification of Spinal Cord Injury; kg, kilogram; m^2^, square meter; n. a., not applicable; SCI, spinal cord injury*.

### The Interrater Reliability of Sharp/Dull Discrimination Differs Between Dermatomes With Intact and Altered Sensation

We determined the reliability of sharp/dull discrimination in dermatomes of non-disabled controls, differentiated between dermatomes AT/ABOVE and BELOW the sensory level in the SCI group, and compared the interrater reliability of intact dermatomes of non-disabled participants with those of SCI participants AT/ABOVE the sensory level.

The mean of correct responses per 12 stimuli was 11.75 ± 0.61 (mean ±*SD*) in the non-SCI group and 11.27 ± 1.14 (AT/ABOVE) and 5.68 ± 4.70 (BELOW) in the SCI group (overall 7.69 ± 4.67 in the SCI group). Individuals in the non-SCI group could correctly discriminate in 98.62% of all tested dermatomes averaged over all tools and examiners. In the SCI group, individuals were able to correctly discriminate between sharp and dull in 92.55% of dermatomes AT/ABOVE and in 31.20% BELOW the sensory level.

Notably, the percentage agreement for the three raters differed between the non-SCI group (97.33%) and intact dermatomes AT/ABOVE of individuals with SCI (89.20%). This implies a false-negative rate of 2.67% in dermatomes of non-SCI and 10.80% in intact dermatomes AT/ABOVE in the SCI group. In individuals with SCI, the interrater reliability was substantial for all tested dermatomes (κ 0.68; CI 0.679–0.681) and moderate in the segments BELOW (κ 0.54; CI 0.539–0.543) the sensory level. Table 2 illustrates κ coefficients (all p < 0.01) and agreement rates for all groupings.

**Table 2:** Interrater reliability for three raters in individuals with and without spinal cord injury considering the sensory level and the applied tool.

**Individuals**	**Dermatomes**	**Grouping**	**Fleiss κ**	**95% CI**	**% agreement**
With SCI	All	All tools	0.680	0.679 to 0.681	83.97
	AT/ABOVE SL	All tools	0.217	0.215 to 0.220	89.20
		Safety pin (4 cm)	0.097	0.092 to 0.102	87.14
		Safety pin (5 cm)	−0.020	−0.025 to −0.015	96.15
		Neurotip^®^	0.259	0.254 to 0.264	82.05
		Transofix^®^	0.218	0.213 to 0.223	89.74
		Cottontip	0.251	0.246 to 0.256	90.93
	BELOW SL	All tools	0.541	0.539 to 0.543	80.94
		Safety pin (4 cm)	0.642	0.638 to 0.646	84.78
		Safety pin (5 cm)	0.526	0.522 to 0.529	79.78
		Neurotip^®^	0.553	0.549 to 0.556	84.89
		Transofix^®^	0.436	0.432 to 0.440	76.37
		Cottontip	0.534	0.531 to 0.538	78.94
Non-SCI	All	All tools	0.021	0.020 to 0.022	97.33
		Safety pin (4 cm)	−0.002	−0.005 to 0.001	99.53
		Safety pin (5 cm)	−0.007	−0.010 to −0.004	98.57
		Neurotip^®^	−0.024	−0.027 to −0.021	95.24
		Transofix^®^	−0.005	−0.008 to −0.002	99.05
		Cottontip	0.047	0.044 to 0.050	94.29

*Shaded boxes denote that Fleiss κ is not interpretable due to a ceiling effect (35), p < 0.01 for all κ in white boxes. cm, centimeter; SL, sensory level; SCI, spinal cord injury; 95% CI, 95% confidence interval*.

**Figure 2:**
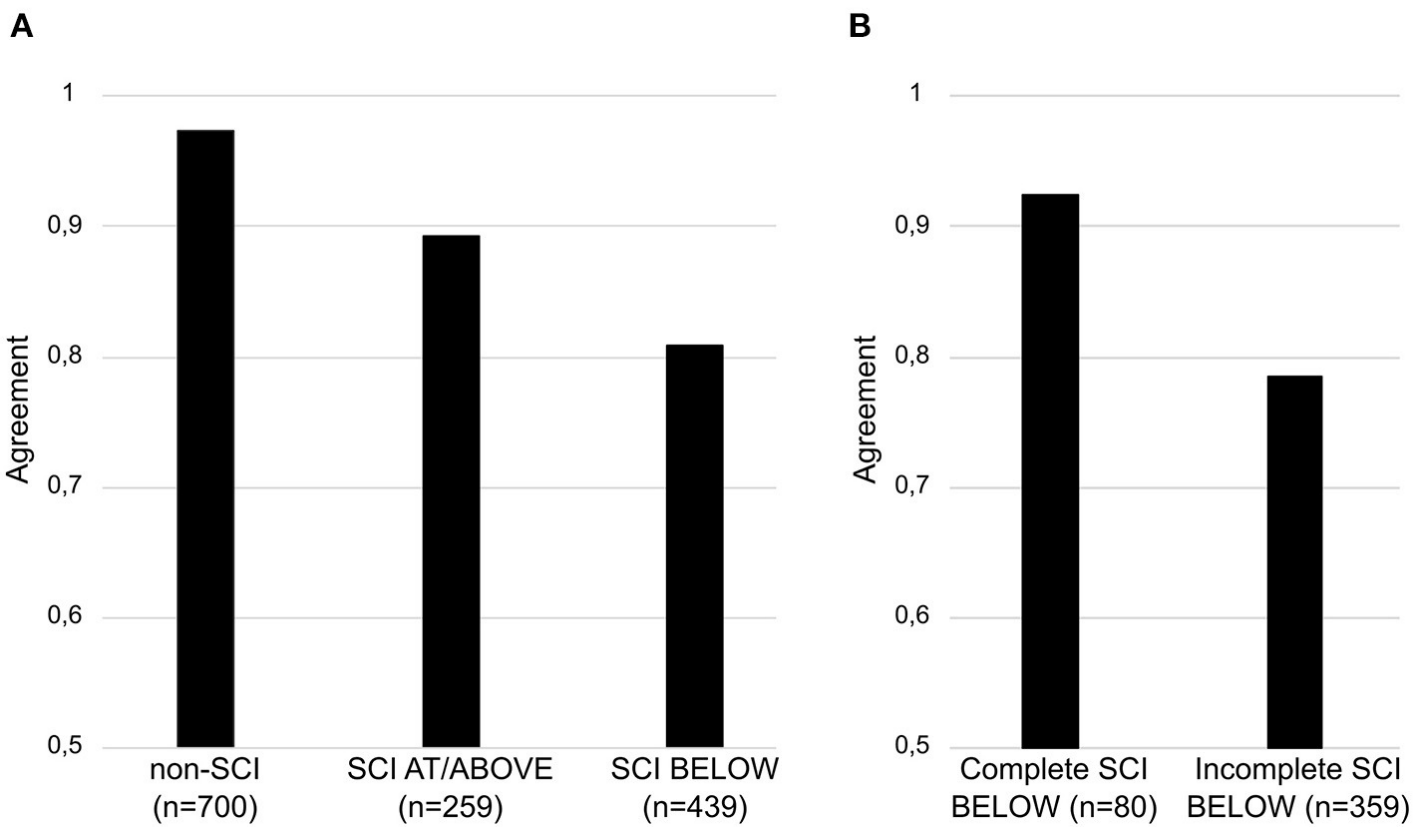
Comparative results of percentage agreement for three raters. The results are illustrated for individuals without and with spinal cord injury **(A)**. In participants with spinal cord injury, results are illustrated for dermatomes AT/ABOVE and BELOW **(A)** the sensory level as well as for complete and incomplete lesions BELOW **(B)** the sensory level. The total number of dermatomes examined by three raters is stated as “*n*”. Agreement (vertical axis) is presented as decimal fraction of the percentage. SCI, spinal cord injury; SL, sensory level.

To allow for comparison of our results with those of other studies (15, 17), we separately determined the interrater reliability of complete [ASIA Impairment Scale (AIS) A] and incomplete lesions (AIS B, C, D). Accordingly, we found a better agreement in dermatomes BELOW of complete as compared to incomplete lesions (93 vs. 78%). Figures 2A,B illustrate the agreement rates for all groupings.

### Minor Effect of Different Examination Tools on Interrater Reliability

According to the survey within EMSCI (23), a variety of tools are administered for sharp/dull discrimination. To identify the most reliable, we compared the interrater reliability coefficients of five representative tools. In the non-SCI group, the Transofix^®^ and both safety pins showed an agreement >98.0%, whereas the Neurotip^®^ and the cotton tip achieved an agreement above >90.0%. In dermatomes AT/ABOVE, the safety pin (5 cm) achieved the highest agreement (96.2%). The agreement of the remaining tools reached from 82.7% for the Neurotip^®^ to 90.9% for the cotton tip.

In dermatomes BELOW the sensory level, only the safety pin (4 cm) yielded a substantial interrater reliability (κ 0.64; CI 0.638–0.646). All other tools revealed a moderate reliability (range κ 0.44–0.55).

### An 80% Correct Response Rate Is Appropriate for Accurate Sharp/Dull Discrimination and Reduces the Risk of Guessing

ISNCSCI recommends a threshold of 80% as standard for intact sharp/dull discrimination (eight correct responses out of 10 stimuli). This threshold is currently based on the examiner's clinical judgment and the objective to reduce the probability of guessing (11). Aiming to verify this approach, the dichotomization was systematically analyzed in the SCI group for all possible thresholds for dermatomes BELOW the sensory level. The kappa coefficients as well as the agreement were additionally considered (Figure 1).

All cutoff values below nine showed a probability of guessing of more than 10% and were therefore not further evaluated. The cutoff value of nine correct responses is the most reasonable trade-off between guessing probability (7.3%), reliability (moderate κ = 0.63), and agreement (82.44%). Such a cutoff (9 out of 12) represents a formal correct response rate of 75%.

## Discussion

By evaluating the interrater reliability of sharp/dull discrimination in a cohort of both individuals with SCI and non-disabled participants, we found conclusive reliability of this fundamental part of the pin-prick examination for testing the integrity of spinothalamic tract function/pain perception (36, 37). This is further emphasized by consistently narrow confidence intervals of the reliability coefficients (31).

Referring to the ongoing discussions on the psychometric properties of the whole pin-prick examination, we found for the sharp/dull discrimination that (1) sensory integrity does indeed have an impact on its reliability, whereas (2) different examination tools did not have a major influence and (3) an 80% correct response rate appears to be reasonable for reliable determination of a clinically largely intact function of the spinothalamic tract. However, it has to be pointed out that the approach presented did not consider the grading of pin-prick sensation as the more subjective part of the pin-prick examination. Indeed, ISNCSCI recommends evaluating spinothalamic tract function on a three-point scale for a more nuanced grading of its integrity (0 = absent sharp/dull discrimination; 1 = intact sharp/dull discrimination but altered pin-prick sensation; 2 = intact sharp/dull discrimination and normal pin-prick sensation). According to that, the examiner firstly explores whether the participant can correctly discriminate between randomly applied sharp and dull stimuli (i.e., differentiation between grade 0 vs. grade 1 or 2). In the second part, the quality of the pin-prick sensation is tested in reference to an unimpaired skin area, preferably on the cheek, to differentiate between grades 1 and 2 (11). Although not in full accordance with the pin-prick examination of ISNCSCI, the presented study design yet facilitates a more specific interpretation for the routinely used neurological sharp/dull examination for spinothalamic tract function (38).

The very low false-negative rate in non-disabled controls underlines the foundation of sharp/dull discrimination as a suitable assessment to be applied to individuals with impaired spinothalamic tract function. As expected, this finding was confirmed with corresponding results in intact dermatomes of individuals with SCI AT/ABOVE the sensory level, albeit slightly worse results regarding the false-negative rate in the SCI cohort. The underlying causes of this remarkable difference between non-disabled individuals and those with SCI might be due to numerous reasons, such as drugs that potentially act on the central nervous system or a significant higher age of the SCI group but could also be related to structural or functional changes in the central nervous system after SCI. An accurate identification of these potential influencing factors and a precise evaluation of their impact on the sensory perception is an important aspect of further own research. The consideration of segments in relation to the sensory level confirmed the preceding assumption that reliability may be overestimated in evaluation of sum scores. Accordingly, the found percentage agreement of sharp/dull discrimination was superior AT/ABOVE as compared to BELOW the sensory level in participants with SCI.

Our cohort, which shows a distribution of clinical characteristics, such as the lesion level and severity, comparable to published data (39), showed higher agreement rates BELOW the sensory level in the subgroup of complete (98%) compared to incomplete (78%) lesions. This was most probably based on a higher number of dermatomes with totally absent sensory function in complete lesions compared to incomplete lesions with a higher number of dermatomes having preserved sensory function (11). This finding complements previous studies (17, 33). In any case, the results indicate that clinicians should take care when examining patients with SCI below the level of injury, particularly when lesions are (sensory) incomplete.

When focusing on the commonly applied tools for sharp/dull discrimination, all investigated instruments yielded reasonable results. The safety pin is officially endorsed to be used in ISNCSCI (11) and easily accessible. The medium-sized safety pin (4 cm) yielded the comparatively highest reliability. The remaining instruments revealed moderate results BELOW and agreements >80% AT/ABOVE the sensory level. However, it has to be noted that all examiners in this study were trained in ISNCSCI and were regularly using the medium-sized safety pin (4 cm). Thus, the high experience with this tool may have led to a bias toward higher reliability for the safety pin. This fact notwithstanding, all examiners reported issues with the handling of various used tools, which may also have contributed to the observed differences in reliability.

Regarding the threshold on the required number of correct responses for determining an intact sharp/dull discrimination, it appears expedient to target the highest possible reliability with a simultaneously low probability of guessing. Considering this, we could confirm that the correct response rate of 80% recommended by ISNCSCI for a clinically intact sharp/dull discrimination appears to be adequate. Specifically applied to our study design, this was reflected by a rate of nine correct responses out of 12 repetitions, resulting in a theoretical threshold of 75% correct responses. However, an implementation in both a rigid research setting and a clinical routine depends on broad acceptance among potential users. This, in turn, is only realistic if such examination techniques are catchy, easy to use, and rapid to apply. Thus, we recommend retaining the already established approach according to ISNCSCI. This requires a rather conservative approach, with the correct response rate determined here as optimal being raised from 75 to 80%. The maximum number of 10 repetitions per dermatome in ISNCSCI would remain unaffected, though.

The presented results complement previous studies in both pediatric (16, 17) and adult (18, 33) individuals with SCI. These found at least moderate reliability for the pin-prick examination including the sharp/dull discrimination. Related factors that might explain differences to our results comprise different study characteristics, such as pediatric/juvenile cohorts (15–17) and different examination tools (18). Furthermore, these studies determined interrater reliability for total scores of the pin-prick examination and did not take characteristics of intact and altered dermatomes into account (16, 17, 31, 33). One group (17) at least determined segmental reliability of dermatomes and myotomes separately but did not differentiate between AT/ABOVE and BELOW the sensory level.

In summary, reliability does not guarantee for validity. However, the proof of reliability is a fundamental prerequisite for validity (40). This also applies to the pin-prick examination as a commonly used assessment (38), although all facets of psychometric properties (12) have to be considered when deciding on accurate thresholds for assessing the sharp/dull discrimination. Referring to the implication of this study, a clear statement can be inferred for an accurate sharp/dull discrimination: the used instrument is less important than dermatome integrity. Specifically, the repeated examination of the dermatomes BELOW the sensory level with potentially preserved function is crucial to ensuring reliable results and to avoiding an undue guessing probability.

When facing a study situation, comparable to an exam situation in school or university, individuals could be tempted to competitive behavior and divergent responses (Hawthorne effect) (27). This underlines the need of examination techniques that are as objective as possible. Considering this, the sharp/dull discrimination examination as implemented in this study is a largely objective approach to evaluate the spinothalamic tract function, albeit representing a streamlined version of the pin-prick examination of ISNCSCI (6, 11).

Hence, the sharp/dull discrimination examination might also prove as a reasonable and recommendable technique for assessing spinothalamic tract integrity in neurological diseases beyond SCI.

## Study Limitations

In this study, sharp/dull discrimination was tested in seven out of 28 dermatomes. Psychometric properties may vary slightly in the remaining dermatomes. However, we rather intended to focus on interrater reliability regarding the sharp/dull discrimination with different tools on a segmental level. High percentage agreement does not automatically confirm high reliability (29). However, commonly used reliability coefficients are not applicable in parameter distributions showing a prominent ceiling effect (34). Relying on the percentage agreement might limit our findings in intact dermatomes. Nevertheless, it represents the only feasible approach to use a common parameter to compare the results in both groups. Percentage agreement is known to be the most intuitive reliability measure and has been requested by clinicians to accompany abstract reliability coefficients (19).

## Conclusion

The ability of sharp/dull discrimination is a reliable measure for evaluating spinothalamic tract function in adults, when performed by trained examiners. It might not only be suitable for individuals with SCI, but also represents a reasonable easy-to-apply clinical bedside test, which can be of use in a number of neurological disorders with accompanying sensory dysfunction. All tested instruments are reasonable to be considered in clinical practice, if the officially recommended safety pin is not available. A threshold of 80% correct responses out of 10–12 trials for confirmation of a preserved sharp/dull discrimination is most suitable in terms of reliability and guessing probability. Causal attribution of the identified differences in the reliability of sharp/dull discrimination between clinically intact dermatomes of individuals with SCI and unaffected dermatomes of individuals without SCI requires further investigation.

## Data Availability Statement

The raw data supporting the conclusions of this article will be made available by the authors upon reasonable request, without undue reservation.

## Ethics Statement

The studies involving human participants were reviewed and approved by the Ethics Commission Medical Faculty Heidelberg, Heidelberg University, Alte Glockengießerei 11/1, 69115 Heidelberg, Germany. The patients/participants provided their written informed consent to participate in this study.

## Author Contributions

LH contributed substantially to study conception, data analysis, data interpretation and drafted the research article. CS contributed substantially to study conception and data interpretation. He supported the data analysis, the draft of the research article and revised the manuscript. DS, CH, and TK were involved in the data collection. NW and RR revised the research article. SF contributed substantially to study conception and data interpretation. He drafted the research article and revised the manuscript. All authors contributed to the article and approved the submitted version.

## Conflict of Interest

The authors declare that the research was conducted in the absence of any commercial or financial relationships that could be construed as a potential conflict of interest.

## Abbreviations

CR2: correct responses binary
EMSCI: European Multicenter Study about Spinal Cord Injury
ISNCSCI: International Standards for Neurological Classification of Spinal Cord Injury
SCI: spinal cord injury
SD: standard deviation
SL: sensory level.
