# Supplementary material for: Revisiting the Examination of Sharp/Dull Discrimination as Clinical Measure of Spinothalamic Tract Integrity
Source: Front Neurol. 2021 Jul 1;12:677888. doi: 10.3389/fneur.2021.677888 (PMC8280296; doi:10.3389/fneur.2021.677888)
Supplement: Supplementary file 1 [file Image_1.PDF]

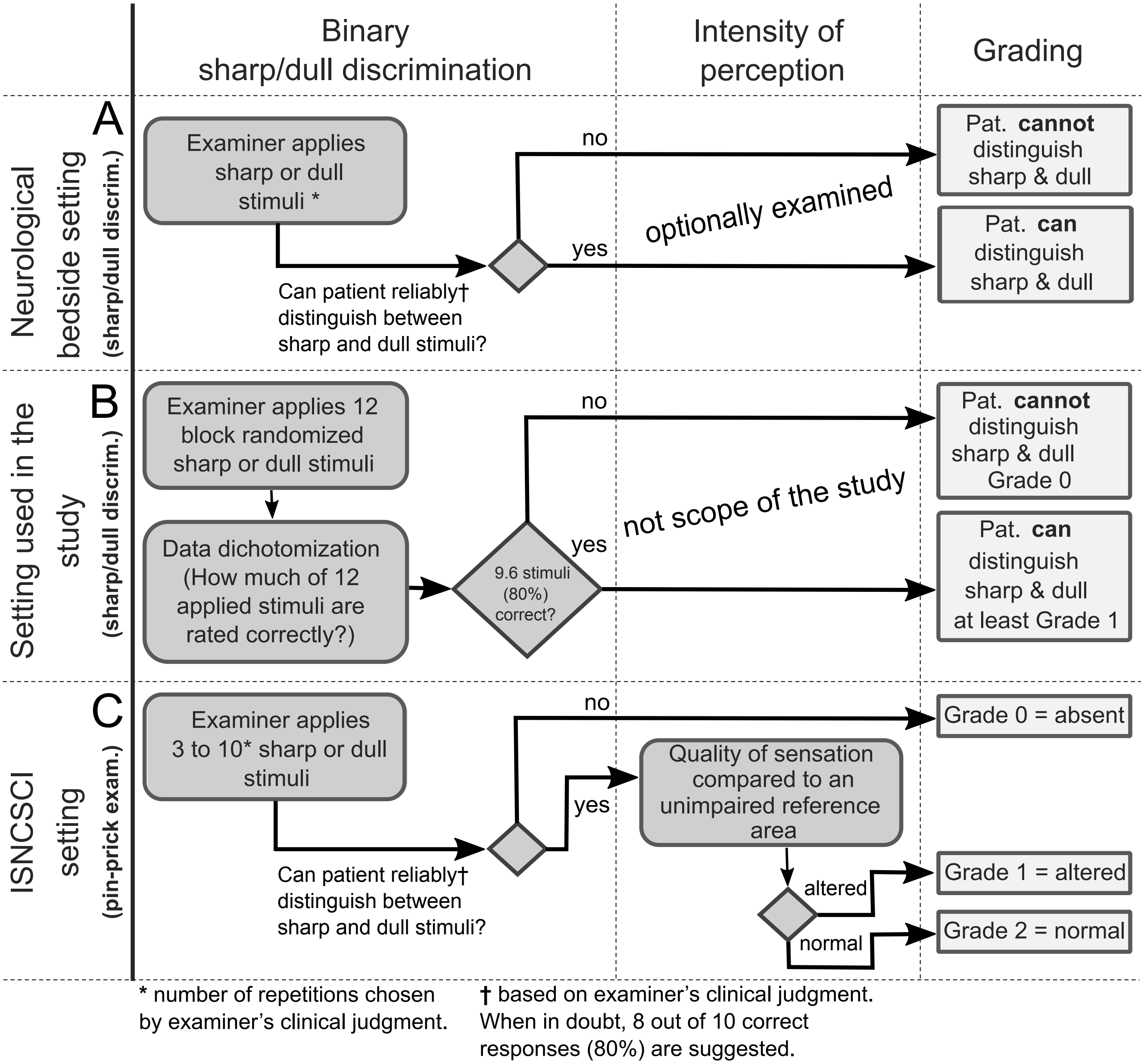

Supplementary Figure 1.

The examination algorithms for neurological bedside setting for examination of spinothalamic tract function (A), the experimental study setup for sharp/dull discrimination (B) and the setting for pin-prick examination (C) according to the International Standards for Neurological Classification of Spinal Cord Injury (ISNCSCI).

In the "Binary sharp/dull discrimination" the examiner explores if the participant can correctly and reliably discriminate between randomly applied sharp and dull stimuli. The ability to discriminate is objectifiable by performing as much repetitions as needed to minimize the probability of guessing the correct result. When in doubt it is stipulated to apply 10 stimuli with a safety pin to reduce the probability of guessing to less than 5%. For the experimental setting, always 12 stimuli were applied. If the participant cannot discriminate, the final grading is 0 for the examined segment.

The second step of examination ("Intensity of perception") is in particular a part of ISNCSCI and should discriminate grade 1 from 2. Accordingly, altered sensation leads to a grade of 1, normal sensation to a grade of 2. The final decision regarding the presence of a changed sharp and/or dull sensation is made subjectively by the participant and the examiner must rely on the answer. It indeed allows a more detailed assessment of sensory and spinothalamic function but may also be biased by the patient and therefore limits the conclusiveness of clinical findings. An objectification of this rating is not possible.
